# The ITIM-Containing Receptor: Leukocyte-Associated Immunoglobulin-Like Receptor-1 (LAIR-1) Modulates Immune Response and Confers Poor Prognosis in Invasive Breast Carcinoma

**DOI:** 10.3390/cancers13010080

**Published:** 2020-12-30

**Authors:** Chitra Joseph, Mansour A. Alsaleem, Michael S. Toss, Yousif A. Kariri, Maryam Althobiti, Sami Alsaeed, Abrar I. Aljohani, Pavan L. Narasimha, Nigel P. Mongan, Andrew R. Green, Emad A. Rakha

**Affiliations:** 1 School of Medicine, The University of Nottingham and Nottingham University Hospitals NHS Trust, Nottingham City Hospital, Nottingham NG7 2RD, UK; mszmst@exmail.nottingham.ac.uk; 2 Division of Cancer and Stem Cells, School of Medicine, University of Nottingham Biodiscovery Institute, University Park, Nottingham NG7 2RD, UK; msxma39@exmail.nottingham.ac.uk (M.A.A.); msxyk2@exmail.nottingham.ac.uk (Y.A.K.); msxma32@exmail.nottingham.ac.uk (M.A.); msxsa33@exmail.nottingham.ac.uk (S.A.); msxaa98@exmail.nottingham.ac.uk (A.I.A.); mzxpl1@exmail.nottingham.ac.uk (P.L.N.); svznpm@exmail.nottingham.ac.uk (N.P.M.); mrzarg@exmail.nottingham.ac.uk (A.R.G.); 3 Department of Applied Medical Sciences, Unayzah Community College, Qassim University, Unayzah 56435, Saudi Arabia; 4 Department of Clinical Laboratory Science, Faculty of Applied Medical Science, Shaqra University 33, Shaqra 11961, Saudi Arabia

**Keywords:** breast cancer, LAIR-1, immune cell markers, prognosis, collagens

## Abstract

**Simple Summary:**

Breast cancer exhibits significant genetic and clinical heterogeneity. Given the importance of understanding tumour-immune interactions to enable the development of novel immunotherapies, identification of novel prognostic biomarkers is important for accurate predictions of the breast cancer patient’s outcome and treatment decisions. The aim of this retrospective study was to assess the potential prognostic value of the leukocyte-associated immunoglobulin-like receptor-1 (LAIR-1), a collagen-binding immunoreceptor tyrosine-based inhibition motifs (ITIM)-bearing inhibitory receptor, that plays an important role in the regulation of the immune system and tumour progression. Our study highlights the importance of LAIR-1 expression and the role of the immune microenvironment in breast cancer progression and worse clinical outcome. Further functional investigation warrants understanding the crosstalk between immune checkpoint blocking agents, immune microenvironment and its underlying mechanisms for targeted therapy development.

**Abstract:**

Background: The leukocyte-associated immunoglobulin-like receptor-1 (LAIR-1) plays a role in immune response homeostasis, extracellular matrix remodelling and it is overexpressed in many high-grade cancers. This study aimed to elucidate the biological and prognostic role of LAIR-1 in invasive breast cancer (BC). Methods: The biological and prognostic effect of LAIR-1 was evaluated at the mRNA and protein levels using well-characterised multiple BC cohorts. Related signalling pathways were evaluated using in silico differential gene expression and siRNA knockdown were used for functional analyses. Results: High LAIR-1 expression either in mRNA or protein levels were associated with high tumour grade, poor Nottingham Prognostic Index, hormone receptor negativity, immune cell infiltrates and extracellular matrix remodelling elements. High LAIR-1 protein expression was an independent predictor of shorter BC-specific survival and distant metastasis-free survival in the entire BC cohort and human epidermal growth factor receptor 2 (HER2)+ subtype. Pathway analysis highlights LAIR-1 association with extracellular matrix remodelling-receptor interaction, and cellular proliferation. Depletion of LAIR-1 using siRNA significantly reduced cell proliferation and invasion capability in HER2+ BC cell lines. Conclusion: High expression of LAIR-1 is associated with poor clinical outcome in BC. Association with immune cells and immune checkpoint markers warrant further studies to assess the underlying mechanistic roles.

## 1. Introduction

The leukocyte-associated immunoglobulin-like receptor-1 (LAIR-1/CD305) is a glycoprotein, which has two immunoreceptor tyrosine-based inhibition motifs (ITIMs), and it is expressed on most immune cells [1,2]. Interaction of LAIR-1 with its cognate binding ligands, such as extracellular matrix (ECM) collagen [3] and the C1q complement component [4] were shown to inhibit immune cell activation [5]. As collagens play a major role in tumour progression, collagen-mediated stimulation of LAIR-1 may activate ITIMs, resulting in the inhibition of immune cell activity [6]. The *LAIR-1* gene maps to 19q13.4, which includes several genes encoding leukocyte immunoglobulin-like receptors reported to have an immunomodulatory effect on a wide range of immune cells [7]. LAIR-1 expression in chronic lymphocytic leukemia (CLL) is associated with disease stage and the proliferation of malignant hematopoietic cells [8,9]. In hepatocellular carcinoma, LAIR-1 expression is positively associated with tumour grade, stage, and worse overall survival [10]. Moreover, tumours overexpressing collagens are associated with poor patient survival and this may be related to collagen-mediated immune cell modulation via LAIR-1 [11].

Previous studies indicate that the immune system plays an important role in many cancers [12], including breast cancer (BC) [13]. Tumour-infiltrating lymphocytes (TILs) are associated with better outcomes in BC, especially in the triple negative tumours (TNBC) [14]. However, the composition of TILs in BC is variable and the association with outcome can be explained through different mechanisms. Lymphocytes can secrete interleukins (ILs) such as IL6, which in turn activates PI3K/AKT, STAT3 signalling, and generates a positive feedback loop between the tumour cells and the immune microenvironment [15]. The prominent presence of CD8+ve T cells within TILS is linked with a better prognosis [16], whereas TILS harboring T cells expressing forkhead box P3 (FOXP3) or programmed cell death 1 (PD-1) are associated with worse prognosis [17]. PD-1/programmed cell death ligand 1 (PD-1/PD-L1) are well known immune checkpoint components that send inhibitory signals to T cells to suppress the anti-tumour response [18,19]. In light of previous studies, ITIM receptors containing biomarkers are best in targeting PD-1/PD-L1 blockade therapies [20].

The expression of LAIR-1 in tumour cells and its association with immune cell function supports a role for LAIR-1 in tumour biology and thus requires further studies. Indeed, the biological role of LAIR-1 in BC has yet to be elucidated. BC is the most common leading cause of cancer-related death amongst women and thus identification of new prognostic markers and therapeutic targets is imperative to progress towards personalised treatment. Thus, this study investigates the association of LAIR-1 expression with commonly recognised clinical and biological variables, total and subtype of immune cells and its prognostic role in BC. In addition, mechanistic functions of LAIR-1 in BC cells in terms of its impact on cell proliferation and invasion are defined using an in vitro model.

## 2. Results

### 2.1. LAIR-1 Expression

Association between *LAIR-1 mRNA* and clinicopathological data of the Molecular Taxonomy of Breast Cancer International Consortium (METABRIC) cohort are summarised in Table 1. High expression of *LAIR-1* mRNA was seen in 46% of cases (910/1980). High *LAIR-1 mRNA* expression was associated with higher histological tumour grade, high NPI, Basal and human epidermal growth factor receptor 2 (HER2) enriched PAM50 subtypes and hormone receptor oestrogen receptor (ER) and progesterone receptor (PgR)) negativity (all *p* < 0.001).

**Figure 1:**
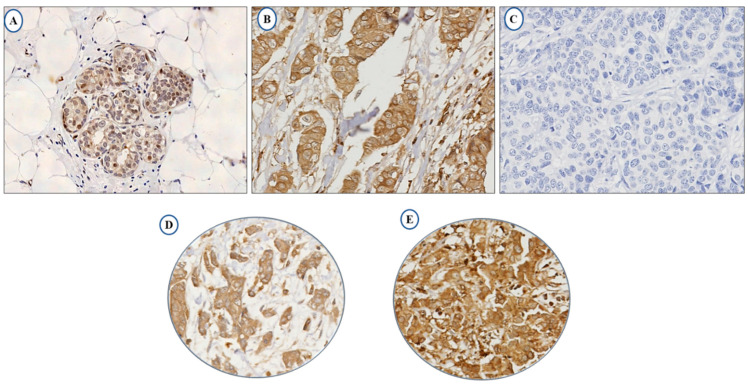
Photomicrographs showing immunohistochemical expression of LAIR-1 in breast cancer: morphological characteristics of LAIR-1 immunohistochemistry in full-face breast cancer tissue (**A**–**C**). Normal terminal ductal lobular unit showing weak LAIR-1 expression (**A**) and (**B**) showing slightly increased immunoreactivity in invasive tumour cells. (**C**) showing the negative control that was performed omitting the primary antibody. LAIR-1 protein expression in breast cancer tissue microarray (TMA) cores showing low (**D**) and high (**E**) immunoreactivity respectively

The full-face tissue sections demonstrate homogeneous expression of LAIR-1 in BC cells, indicating the suitability of tissue microarrays (TMAs) to evaluate its expression. Normal breast terminal ductal lobular units demonstrate weak LAIR-1 cytoplasmic staining (Figure 1A), while tumour cells reveal slightly increased immunoreactivity (Figure 1B) and Figure 1C represents negative LAIR-1 immunohistochemistry (IHC) expression. LAIR-1 IHC expression predominantly shows cytoplasmic staining with occasional membranous staining in a few tumour cells. This was consistent with Wang et al., who found a similar predominant LAIR-1 cytoplasmic expression in human cervical cancer cells [21]. Cytoplasmic LAIR-1 expression varies from weak to strong as shown in Figure 1D,E. LAIR-1 expression did not follow a normal distribution, therefore for dichotomisation into low/high expression, the median H-score of 120 was used and the expression ranged from 0 to 280 H-score. High expression was observed in (H-score ≥ 120) 51% of the cases and (H-score < 120) 49% of tumours displayed negative/low expression.

High LAIR-1 protein expression associated with clinicopathological parameters are summarised in Table 2: such as higher tumour grade, low expression in lobular tumour types (both; *p* < 0.001), high NPI (*p* = 0.012) and estrogen receptor negative status (*p* = 0.013). High LAIR-1 expression was positively associated with Cyclin B1 (*p* = 0.010), c-MYC and Cdc42 (both; *p* < 0.001).

### 2.2. LAIR-1 Correlation with Collagens and Immune Cell Types

**Figure 2:**
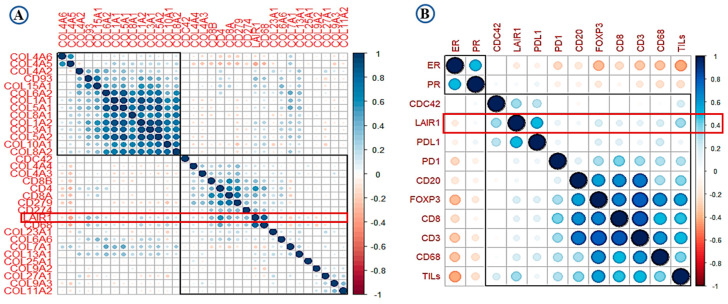
Correlation matrix shows the correlation between LAIR-1, collagens, immune cell markers and tumour-infiltrating lymphocytes (TILs) (**A**) at mRNA level and (**B**) at the protein level. Blue colour refers to positive correlations, while the red colour reflects negative correlations. The intensity of the colour propionate to the correlation coefficient.

High expression of *LAIR-1* showed positive correlation with collagens including collagen I, III, IV, V, VI, VIII, XIII, and XV (*r* > 0.30, *p* < 0.001). Total CD4+, CD68+ and CD8+ cell showed highest correlation with *LAIR-1* at mRNA level (*r* > 0.45, *p* < 0.001). At the protein level, PD-L1, Cdc42 and TILs showed the strongest positive correlation with LAIR-1 (*r* > 0.50, *p* < 0.001). Association between overall TILs, various immune cell subtypes, PD-1, PD-L1, hormonal receptors and LAIR-1 mRNA/protein is summarised in the correlation matrix (Figure 2A,B). This association was confirmed using the Breast cancer Gene Expression Miner (bc-GenExMiner) v 4.5 (Figure S1A–D) and The Cancer Genome Atlas (TCGA) data (Figure S1E–H).

### 2.3. Prognostic Value of LAIR-1 Expression

Patients showing high *LAIR-1* mRNA expression were associated with shorter patient survival in entire BC cohort (HR = 1.2, 95% CI 1.0–1.5, *P* = 0.023, Figure S2A). This association was confirmed using the bc-GenExMiner v 4.5 (DNA microarray data (*p* = 0.032; Figure S2B) and cBio Cancer Genomics Portal datasets (*p* < 0.001; Figure S2C,D).

**Figure 3:**
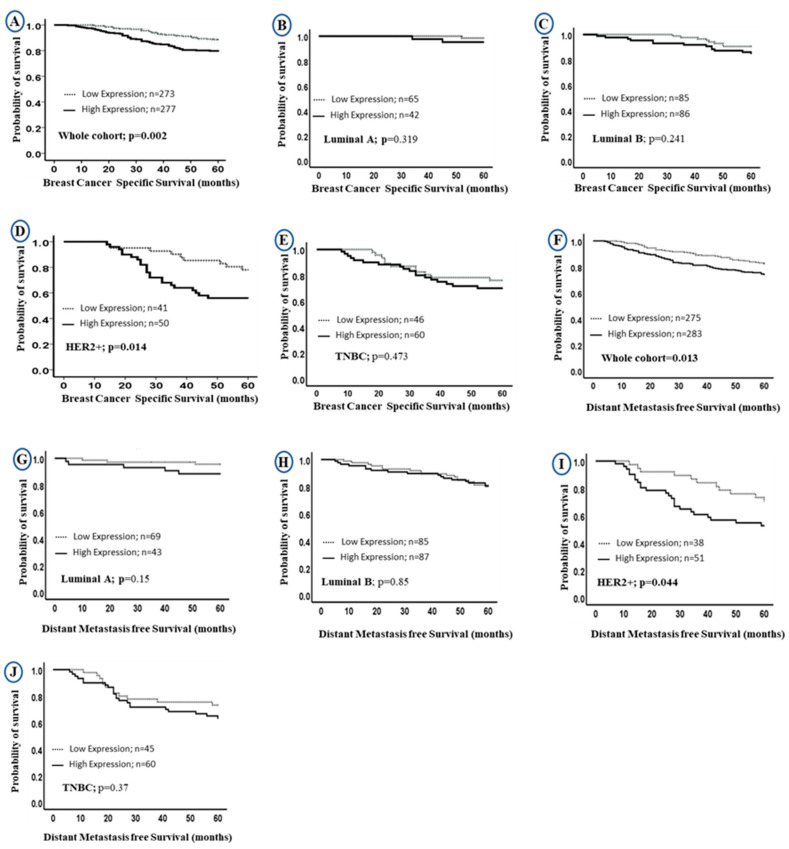
Kaplan-Meier curves of LAIR-1 protein expression and patient outcome: LAIR-1 protein expression and breast cancer (BC) specific survival (BCSS) (**A**) in the whole cohort, (**B**) Luminal A, (**C**) Luminal B, (**D**) HER2+ and (**E**) triple negative tumours (TNBC) BC subtypes. LAIR-1 protein expression and DMFS in (**F**) in the whole cohort, (**G**) Luminal A, (**H**) Luminal B, (**I**) HER2+ and (**J**) TNBC BC subtypes.

Univariate survival analysis revealed that higher LAIR-1 protein expression was associated with shorter BC specific survival (BCSS) in the entire BC cohort (HR = 2.0, 95% CI 1.3–3.0, *p* = 0.002; Figure 3A, HR = 1.6; 95% CI 1.0–2.3; *p* = 0.013; Figure 3F, for BCSS and distant metastasis free survival (DMFS), respectively). When stratified into intrinsic molecular subtypes (Figure 3B–E,G–J), the association between LAIR-1 high expression and shorter survival was maintained in the HER2 enriched class (HR = 2.5, 95% CI 1.1–5.3, *p* = 0.014; Figure 3D and HR = 2.0; 95% CI 1.0–4.1; *p* = 0.044; Figure 3I for BCSS and DMFS, respectively).

Multivariate Cox regression revealed that high LAIR-1 expression was an independent predictor of adverse BCSS and DMFS survival (HR = 2.0, 95% CI 1.1–3.4, *p* = 0.017 and HR = 1.6, 95% CI 1.0–2.5, *p* = 0.046, respectively; Table 3) independent of other confounding prognostic factors including tumour size, tumour grade, stage, CD3+, CD8+, HER2+ status and chemotherapy. When stratified into molecular subtypes, high LAIR-1 expression was significantly associated with poor BCSS (HR = 4.8, 95% CI 1.8–13.0, *p* = 0.003) and shorter DMFS (HR = 5.8, 95% CI 1.9–17.0, *p* = 0.002; Table 3) in the HER2+ subgroup only.

**Figure 4:**
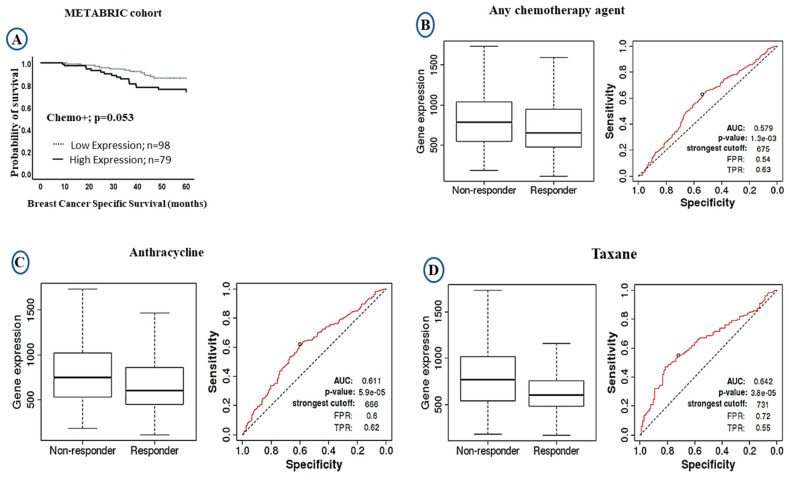
Kaplan-Meier Curves of LAIR-1 expression showing responsive and unresponsive cases to chemotherapy treatment: (**A**) Kaplan-Meier plots of BCSS in the METABRIC cohort showing LAIR-1 mRNA expression in patients who received chemotherapy. The receiver operator characteristic curve (ROC) in response to chemotherapy showing higher LAIR-1 expression was associated with non-responders ‘groups; (**B**) any type of chemotherapy agent, (**C**) anthracycline, and (**D**) taxane.

The LAIR-1 upregulation at both mRNA/protein levels showed significant association with poor clinical outcome. We therefore compared the levels of *LAIR-1* mRNA expression and outcome after stratification of the patients based on chemotherapy treatment. In patients who were given chemotherapy, high *LAIR-1* expression showed a trend of shorter patient survival (HR = 1.0, 95% CI 0.8–1.6, *p* = 0.053: Figure 4A). We further validated the prognostic value of *LAIR-1* expression and its response to chemotherapy using the receiver operator characteristic curve (ROC) plotter. However, patients with high *LAIR-1* expression showed significant association with the non-responders’ group to chemotherapy either to any chemotherapy agent (*p* < 0.001; Figure 4B), or to anthracycline (*p* < 0.001; Figure 4C) and taxane (*p* < 0.001; Figure 4D). In the case of LAIR-1 at protein level there was no significant difference in outcome in patients who received chemotherapy treatment; BCSS (HR = 1.6, 95% CI 0.8–3.6, *p* = 0.197; Figure S3A) and DMFS (HR = 1.3, 95% CI 0.6–2.7, *p* = 0.489; Figure S3B).

### 2.4. LAIR-1 Promotes Cell Proliferation and Invasion Ability in BC Cell Lines

To confirm the role of LAIR-1 in BC behavior and that knockdown of *LAIR-1* can affect BC proliferation and invasion we carried out in vitro experiments. Differential expression of LAIR-1 in BC cell lines revealed high expression in SKBr3 (HER2+) and MDA-MB 231 (TNBC) cell lines (Figure S4A). We have used two independent siRNA targeting LAIR-1 (IDs: s8048 and s8049) relative to a non-targeting scramble (4390843) control siRNA (all purchased from Ambion, ThermoFisher Scientific, Loughborough, UK) to test the efficacy of knockdown. Both siRNA targeting LAIR-1 showed similar knockdown (Figure S4B), so we prioritized one siRNA (s8048) for subsequent functional studies.

**Figure 5:**
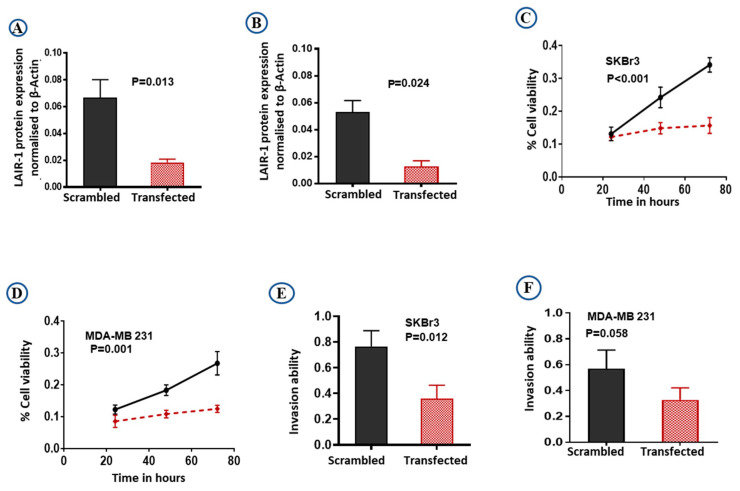
Downregulation of LAIR-1 expression by siRNA transfections. SKBr3 and MDA-MB 231 cells were transfected with scrambled control siRNA or LAIR-1 siRNA. Cells were then seeded in duplicate in corresponding media, total cell extracts were collected 72 h after transfection and 20 µg protein aliquots were blotted with LAIR-1 and β-Actin. Results shown are mean ± standard error of the mean (SEM) of three independent experiments. LAIR-1 protein expression normalised to β-Actin showed more than 95% reduction in the LAIR-1 siRNA cells in both (**A**) SKBr3 and (**B**) MDA-MB 231 cells. Cell proliferation was significantly reduced after LAIR-1 siRNA transfection in both BC cell lines (**C**) SKBr3 and (**D**) MDA-MB 231 as detected by MTS assay. LAIR-1 siRNA transfection showed significantly decreased invasion ability in the SKBr3 cells (**E**), while MDA-MB 231 (**F**) showed a similar trend. Results shown are mean ± standard error of the mean (SEM) from three independent transfections.

The efficiency of knockdown was evaluated by western blotting (Figure S4C,D). *LAIR-1* knockdown expression relative to the β-actin expression was shown to be effective, resulting in almost complete loss of LAIR-1 protein expression in both SKBr3 (Figure 5A; *p* = 0.013) and MDA-MB 231 (Figure 5B; *p* = 0.024) cells. As hypothesised, *LAIR*-*1* knockdown reduced cell proliferation in both SKBr3 and MDA-MB 231 (both *p* < 0.01; Figure 5C,D). Moreover, *LAIR-1* knockdown significantly impaired invasion of SKBr3 cell lines (Figure 5E; *p* = 0.012) with a similar trend observed in MDA-MB 231 cells (Figure 5F; *p* = 0.058).

### 2.5. Pathway Analysis

**Figure 6:**
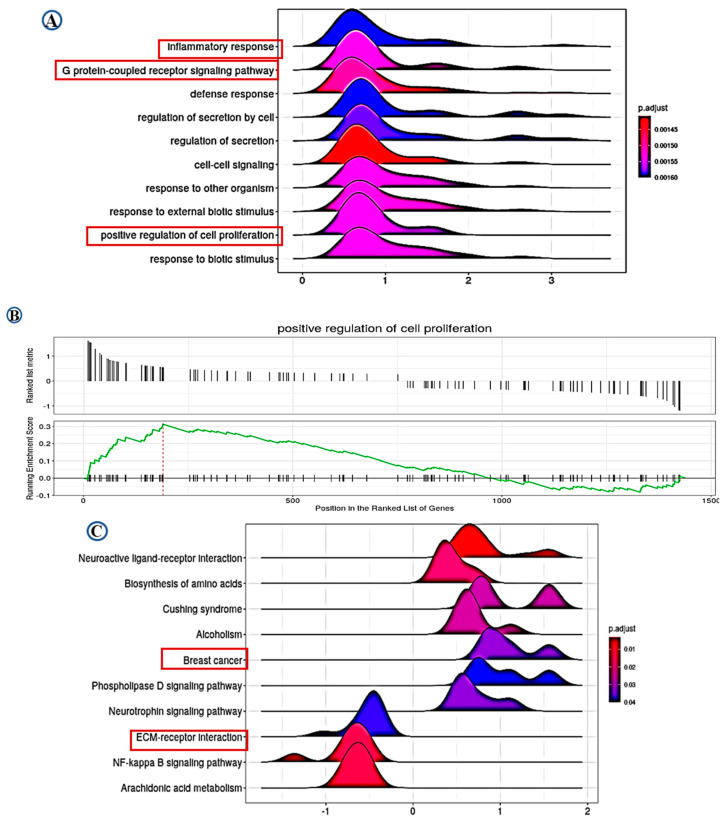
Data of gene ontology (GO) and Kyoto Encyclopedia of Genes and Genomes (KEGG) analysis. Based on the LAIR-1 correlated genes, the enriched information about gene ontology (GO) pathway (**A**,**B**), and the data of KEGG (**C**) were provided, respectively. All pathways mainly highlighting LAIR-1 association with extracellular matrix (ECM)-receptor interaction and positive regulatory role in cell proliferation.

Differential gene expression (DGE) analysis identified 1439 significantly differentially expressed genes associated with LAIR-1 expression, with high LAIR-1 protein expression displaying 773 upregulated and 666 downregulated genes, respectively. Furthermore, applying the over-representation analysis tool (ORA) to perform gene ontology (GO) biological process analysis for DEG associated with upregulated LAIR-1 protein expression were summarised in Figure 6A–C. Gene set enrichment analysis (GSEA) analysis data identified LAIR-1-associated cellular components such as inflammatory response, G protein-coupled receptor signalling pathway and positive regulation of cell proliferation. In addition, GSEA data also revealed an association with LAIR-1 expression and ECM-receptor interaction (Figure 6A,B). Kyoto Encyclopedia of Genes and Genomes (KEGG) pathway analysis further disclosed a series of additional pathways showing strong association with BC, such as Biosynthesis of amino acid pathway, NF-kappa B signalling pathway, Arachidonic acid metabolism pathway, and ECM-receptor interaction pathway (Figure 6C).

## 3. Discussion

Given the importance of understanding tumour-immune interactions to enable the development of novel immunotherapies, new insights into how cancer cells evade the immune system are urgently required. LAIR-1 is a collagen-binding ITIM-bearing inhibitory receptor and plays an important role in the regulation of the immune system. The tumour microenvironment (TME), including ECM-LAIR-1 interaction, may impair anti-tumour immune responses [22]. Despite the proven regulatory role of LAIR-1 in immune cells and the high abundance of collagen molecules in the TME in promoting tumour progression, the potential roles of LAIR-1 are less investigated in BC. Thus, our study has explored the potential prognostic implications of LAIR-1 expression utilising well-annotated multiple BC cohorts. We showed for the first time that LAIR-1 overexpression at the mRNA and protein level is associated with aggressive features of BC and adverse clinical outcome. Collagens are known as LAIR-1 ligands and thereby modulate immune function [3]. The C1q complement molecule has the ability to bind and activate LAIR-1 to evade immune responses and expression of several members of the collagen family, such as collagens I, III, V, VI, XIII, XVII, XVIII, and XXIII [23] is reported to be associated with tumour progression [24,25]. We observed a significant positive association between collagens with high expression of *LAIR-1* mRNA, implying that high expression of collagens by tumour cells may enable these cells to suppress anti-tumour responses via the LAIR-1 immuno-inhibitor. Thus, LAIR-1 represents a mechanistic point of convergence of collagen and immune function.

High expression of LAIR-1 has been reported to be a significant factor in the development of various hematopoietic malignancies [8,9], kidney [26] and ovarian cancers [27]. Consistent with these findings, we revealed that high expression of LAIR-1 at mRNA and protein level is associated with aggressive clinicopathological parameters and proliferation markers. This result matches with the previous study, that LAIR-1 overexpression augmented cell proliferation and silencing of *LAIR-1* significantly inhibited cell proliferation in renal cell carcinoma cells [26]. Moreover, in our study silencing of *LAIR-1* decreased cell proliferation and cell invasion in aggressive BC cell lines. Our pathway analysis data further strengthen the positive association of LAIR-1 and cell proliferation. Altogether, these findings suggest a potential oncogenic role for LAIR-1 expression in breast tumour cells.

High expression of LAIR-1 was significantly associated with shorter patient survival in terms of BCSS and DMFS in the whole cohort and in the HER2+ BC subtypes. The association with the highly proliferative BC, such as HER2 enriched tumours, could be due to the increased immunogenicity of these tumours and increased presence of neoantigens [28]. We have previously demonstrated a strong association between HER2 over-expressing tumours and c-MYC positivity, and this was linked with a poor patient outcome. High c-MYC expression was also associated with an increase in cell cycle activity such as high expression of Cyclin B1 and Ki67 [29]. In vitro and in vivo xenograft studies have confirmed the pro-tumourigenic role of Cdc42 that may stimulate BC proliferation, migration, and metastasis [30]. LAIR-1 strong association with c-MYC and Cdc42, as we observed in our study, and the concomitant high expression of LAIR-1 in these tumours, suggest that these tumours are highly proliferative and are linked with poor prognosis. This is consistent with a previous study by Xu et al., which demonstrated that high LAIR-1 expression is associated with poor survival in brain, colon, kidney and ovarian cancers [22]. In line with our findings, LAIR-1 knockdown significantly downregulated proliferation and invasion capabilities in HER2+ BC cell lines. Altogether, these findings identify an association between LAIR-1 and HER2+ expression and suggest a regulatory role which might result in tumour proliferation/invasion leading to a poor patient outcome.

TILS, CD3+ and CD8+ cell were associated with prognostic value in several malignancies, including BC [31]. Both FOXP3+ and PD-L1+ T cells are associated with poor patient outcome in BC [32,33]. Indeed, our results demonstrated a positive association with immune cell markers (CD8+, CD68+, and PD-L1+ cell) and LAIR-1 at both mRNA and protein levels. Tumour cells are believed to block normal immune regulatory mechanisms. LAIR-1 is reported to inhibit T-cell receptor-mediated signals, via signalling through ERK and by activating protein tyrosine phosphatases [26]. The LAIR-1 with ITIM motifs have been shown to inhibit signalling from immunoreceptor tyrosine-based activation motif (ITAM) containing receptors; the role of these receptors in tumour development is well documented [34]. Increased collagen expression in primary BC is associated with tumour progression, extracellular matrix stiffening and patient mortality [35]. Both C1q and/or collagens binding with LAIR-1 play a major role in suppressing T cell immune responses and may result in the suppression of various immune cells. Our pathway analysis revealed that the G Protein-coupled receptor signalling pathway and ECM-receptor interaction may be associated with essential mechanisms of LAIR-1 in the pathogenesis of BC, identifying a methodical angle for further investigation. These studies suggest that the ability of LAIR-1 to promote an immunosuppressive microenvironment may reflect its association with poor patient survival.

Patients with high expression of *LAIR-1 mRNA* showed association with failure to respond to relevant chemotherapy agents. But at the protein level, there was no significant association with outcome in patients who received chemotherapy treatment. Our results suggest that LAIR-1 could be a potential biomarker that can be added to other established clinical and pathological biomarkers to predict the response of chemotherapy in the candidate patients. However, further work is required to investigate the mechanistic basis of this distinction between LAIR-1 protein and mRNA expression association with chemotherapy response. To further validate our findings, pre-clinical and clinical studies are required to investigate the prognostic value of LAIR-1 and treatment regimes. Targeting phagocytosis checkpoints may complement existing T cell immune-checkpoint inhibitors to maximise anti-tumour responses. PD-1/PD-L1 blockade monotherapy [36,37] in combination with chemotherapy showed a positive outcome in metastatic BC [38,39]. Overall, these findings suggest that blocking LAIR-1-mediated immune suppression in combination with other immune checkpoint blocking agents could have implications for the treatment regimen in combination with conventional therapies.

While this study introduces interesting findings related to LAIR-1 in BC, there are some limitations. These include modest differences in results obtained from gene expression datasets and protein expression which may be attributable to differences in demographic and molecular subtypes’ distributions between tested cohorts. Differences in sample preparation, analysis and interpretation between microarray and RNAseq studies are well established [40,41]. Reassuringly, our study showed a similar significant association of LAIR-1 expression and poor prognostic characteristics in both datasets. Our study is based on a retrospectively collected cohort, so a well-designed randomised clinical trial where patients are treated uniformly is recommended for independent assessment of the expression of LAIR-1. However, our in vitro data suggests the potential pro-tumorigenic role for LAIR-1 in BC. Further, in vitro and/or in vivo studies looking into the potential differential gene expression/pathways on LAIR-1 knock down cells are warranted.

## 4. Materials and Methods

### 4.1. LAIR-1 mRNA Expression

The Molecular Taxonomy of Breast Cancer International Consortium [METABRIC; *n* = 1980] [42], Breast cancer Gene Expression Miner (bc-GenExMiner) version 4.5 (http://bcgenex.centregauducheau.fr; *n* = 4904) [43] (METABRIC and/or TCGA data were excluded from these analyses), and cBio Cancer Genomics Portal datasets [44], were used to explore the clinical and prognostic value of *LAIR-1* mRNA. The ROC plotter data portal was used to evaluate the predictive potential of *LAIR-1* expression and response to chemotherapy [45]. In addition, the data of gene ontology (GO) enrichment analysis, Kyoto Encyclopedia of Genes and Genomes (KEGG) pathway analysis and Gene Set Enrichment Analysis (GSEA) profiles were also accessed.

### 4.2. LAIR-1 Protein Expression

#### 4.2.1. Study Cohort

A large, well-characterised cohort of patients presented with operable invasive BC (*n* = 569) and treated at Nottingham City Hospital, Nottingham, United Kingdom (UK), as previously described [46], was used in this study. Patients’ management was uniform and based on tumour characteristics as defined by the Nottingham Prognostic Index (NPI) and hormone receptor status as previously reported [47]. Hormonal receptor status including oestrogen receptor (ER) and progesterone receptor (PgR) was available and the positive status was defined as those tumours with ≥1% immunoreactivity [48,49]. The assessment of HER2 status was carried out using immunohistochemistry and a chromogenic in situ hybridization technique, to evaluate the gene amplification for the cases with borderline (+2). The definition for HER2 positivity was ≥10% of tumour cells showing intense staining of their membranous (score +3) [48]. Clinical data, tumour characteristics and information on therapy and outcomes are prospectively maintained. The clinical outcome data, including BC specific survival (BCSS), the time (in months) from the date of the primary surgical treatment to the time of death from BC, and distant metastasis free survival (DMFS) was defined as the time interval (in months) from the time of primary surgery to the first occurrence of distant metastasis, were maintained on a prospective basis. BC molecular subtypes were defined based on the IHC profile, including luminal A [ER+/HER2−; Ki67 < 10%], Luminal B [ER+/HER2−; Ki67 ≥ 10%], HER2-positive class [HER2+ regardless of ER status], and TNBC [ER−, PR− and HER2−] [50].

#### 4.2.2. Immunohistochemistry (IHC)

The specificity of LAIR-1 [rabbit polyclonal antibody; Novus Biologicals/R&D Systems; NBP1-84590, UK] was verified by western blot (WB) analysis of whole cell lysates using MDA-MB-231, MCF-7 and, SKBr3 cell lines [obtained from the American Type Culture Collection; Rockville, MD, USA] at 1:750 dilution of the primary antibody. Mouse monoclonal primary antibody β-actin (Sigma-Aldrich, East Yorkshire, UK) was used at 1:5000 dilution as a loading control with molecular weight (~42 KDa). Fluorescent secondary antibodies at [1:15,000] [IR Dye 800CW donkey anti-rabbit and 680RD donkey anti-mouse, LI-COR Biosciences, Lincoln, UK] were used as previously published [51]. The specificity of the antibody was validated by the presence of a single band at ~70 KDa (Figure S5). To evaluate the pattern of LAIR-1 protein expression prior to staining of tissue microarrays (TMAs); full face BC tissue sections (*n* = 10) were selected, based on different tumour grades and histological types and stained. TMAs were previously prepared using a TMA Grand Master^®^ (3D HISTECH^®^, Budapest, Hungary) [46].

For immunohistochemistry, citrate-heat induced antigen retrieval was performed (pH 6.0 at 1000 W for 20 min using a microwave). Expression of LAIR-1 protein was detected using the Novocastra Novolink ^TM^ Polymer Detection System kit (Code: RE7280-K, Leica, Biosystems, Milton Keynes UK), where 4-µm sections were incubated for 60 min with LAIR-1 (rabbit polyclonal antibody; Novus Biologicals/R&D Systems; NBP1-84590, Abingdon UK at dilution 1:500). A negative control, omitting the primary antibody, was carried out. Cytoplasmic LAIR-1 expression was accessed utilising a modified histochemical score (H-score) [52] for the semi-quantitative analyses of immunoreactivity. Briefly, it is a visual approach taking into consideration the intensity of staining and the percentage of stained cells within each tissue core. The staining intensity ((0 (negative), 1 (weak), 2 (moderate), 3 (strong)) multiplied by the percentage (0–100%) for each intensity of representative cells in the tissue, ranging from 0 to 300. The H score is considered a reliable tool that can reflect any heterogeneity of proteins’ expression in the tumour cells as it represents the amount of protein expression (intensity) and the proportion of cells showing such expression (percentage). The scoring was performed blinded to patients’ clinicopathological and outcome, with a subset of cores (~10%) scored independently by another scorer with an interclass correlation coefficient; ICC = 0.9, achieved. Moreover, the discordant cases were re-scored by both observers and a consensus score were agreed and assigned.

To further understand the molecular and clinical significance of LAIR-1 in BC, and integrative analysis of immunohistochemistry data obtained from the same cohort for TILs as assessed on H&E slides [53], immune cell markers (CD8 [54], CD68 [55], CD71 [56], and FOXP3 [32], Cell Division Cycle 42 (Cdc42) [57], proliferation markers (Ki67 [58], epidermal growth factor receptor (EGFR) [59], PIK3CA [60] and Myc proto-oncogene protein (c-MYC) [29] was included in this study.

#### 4.2.3. In Vitro Studies

BC cell lines were chosen based on our proteomic and transcriptomic results, which showed high expression of LAIR-1 in TNBC and HER2+ enriched subtypes. All BC cell lines were purchased from American Type Culture Collection (ATCC, Manassas, VA USA) and cultured as per ATCC recommendations. SKBr3 (McCoy; M9309; Sigma, UK) and MDA-MB 231 (RPMI-1640; D5796; Sigma, UK), all supplemented with 10% fetal calf serum (FBS). Cell lines obtained from ATCC were used within few passages (all; between passage 3–10) from the original stocks, cells was confirmed mycoplasma free (CUL001B; R&D Systems, UK) prior to experiments. Cells were maintained in a 37 °C humidified incubator with 5% carbon dioxide.

Transient (siRNA) knockdown (KD) was performed on MDA-MB 231 and SKBr3 cells with *LAIR-1* siRNA or scrambled negative control siRNA (Silencer^®^ Select siRNA (from Ambion^®^), ThermoFisher Scientific). In a 24-well plate, 5 × 10^4^ cells were seeded per well and transfected with 25 pmol siRNA by using the reverse transfection method with Lipofectamine™ RNAiMAX Transfection Reagent (13778150; ThermoFisher Scientific, UK) as per the manufacturer’s protocol. Cell lysates were collected in RIPA buffer (89900; ThermoFisher Scientific, Loughborough, UK) supplemented with phosphatase inhibitor cocktail and protease inhibitor cocktails (Sigma, UK) and efficiency of transfection were confirmed by western blotting.

The effect of *LAIR-1* knockdown on proliferation was assessed by the (Promega, (G3580); CellTitre 96 Aqueous One Solution Cell Proliferation Assay) 3-(4,5-dimethylthiazol-2-yl)-2, 5-diphenyltetrazolium (MTS) assay. In brief, the control and knockdown cells were seeded at 3000 cells/well in 96-well plate at 37 °C in a 5% CO2 incubator. Proliferation was measured at 24, 48, and 72 h by adding MTS reagent to the wells as per the manufacturer’s protocol. The plates were incubated for 1 h and the absorbance of each well was measured using a Synergy™ 4 (BioTek Instruments, Winooski, VT, USA) at 490 nm.

CytoSelect 24-Well Cell Invasion Assay (Basement Membrane, Colorimetric) from Cell Biolabs (CBA-110 San Diego, CA, USA) was used to assess cell invasion. Cells were incubated overnight in serum-free medium before detaching and seeding in matrix-coated trans-wells. The cells were treated with media and 10% FBS for 24 hrs. The cells in the top chamber were removed and treated with cell stain solution (10 min) and extraction solution per manufacturer’s instructions and measured OD at 560 nm.

#### 4.2.4. Statistical Analysis

Statistical analysis was performed using SPSS, version 24 (Chicago, IL, USA). The median was used to determine cut off points to categorise mRNA (6.01) and protein (H-score = 120) expression levels into low and high subgroups. The Chi-square-test was used to evaluate the association between LAIR-1 mRNA/protein and clinicopathological parameters. Pearson correlation coefficient test was used to assess the correlation between continuous normalised data. For the continuous variables, differences between three or more groups were assessed using one-way analysis of variance (ANOVA) with the post-hoc Tukey multiple comparison test.

Patient univariate survival analysis was evaluated with Kaplan-Meier analyses. Multivariate Cox regression model was used to evaluate the independent prognostic significance of LAIR-1. For all tests, a two-tailed *p*-value < 0.05 was considered as statistically significant. Gene expression was analysed using the subset of Nottingham series included in the METABRIC (*n* = 107) where immunohistochemistry scores were available to investigate the molecular biology underlies LAIR-1 protein expression as an end point. Differential gene expression (DGE) analyses were calculated using the Robina implementation of the Edge-R statistical tool and DGE between high LAIR-1 protein expression (*n* = 37) and low/negative LIAR1 protein expression (*n* = 70) with ≥2 fold-change, and adjusted *p* values < 0.05 were considered significant. and gene set enrichment analysis (GSEA) using the cluster Profiler package in R was used to annotate the DGE list for identification of over-represented gene ontologies and pathways [61].

## 5. Conclusions

This study highlights the importance of the LAIR-1 and the immune microenvironment in BC progression. LAIR-1 expression was significantly associated with poor patient outcome. Additional mechanistic studies to elucidate the crosstalk between immune checkpoint blocking agents, immune microenvironment and LAIR-1 are warranted.

## Figures and Tables

**Table 1:** Associations between leukocyte-associated immunoglobulin-like receptor-1 (*LAIR-1)* mRNA expression and clinicopathological variables in the Molecular Taxonomy of Breast Cancer International Consortium (METABRIC) breast cancer cohort.

Clinicopathological Criteria	*LAIR-1* mRNA Expression N (%)		χ2 (*p* Value)
	Negative/Low Expression	High Expression	
**Age at Diagnosis**			
≤50	234 (55.0)	190 (45.0)	0.091 (0.764)
>50	846 (54.0)	710 (46.0)	
**Tumour Size (cm)**			
≤2.0	488 (57.0)	370 (43.0)	3.280 (0.070)
>2.0	581 (53.0)	520 (47.0)	
**Tumour Stage**			
I	290 (58.0)	211 (42.0)	17.033 **(0.001)**
II	417 (51.0)	408 (49.0)	
III	53 (45.0)	65 (55.0)	
**Histological Grade**			
Grade 1	112 (66.0)	58 (34.0)	56.994 **(<0.001)**
Grade 2	479 (62.0)	291 (38.0)	
Grade 3	435 (46.0)	517 (54.0)	
**Tumour Type**			
Ductal	926 (54.0)	775 (46.0)	11.279 **(0.024)**
Lobular	92 (62.0)	55 (38.0)	
Other special types	29 (60.0)	20 (40.0)	
Mixed tumours	12 (57.0)	9 (43.0)	
**Nottingham Prognostic Index groups**			
Good	438 (64.0)	242 (36.0)	50.501 **(<0.001)**
Moderate	564 (51.0)	537 (49.0)	
Poor	78 (39.0)	121 (61.0)	
**PAM50 Subtype**			
Luminal A	478 (67.0)	240 (33.0)	81.734 **(<0.001)**
Luminal B	254 (52.0)	234 (48.0)	
Basal	127 (39.0	202 (61.0)	
Her2 enriched	116 (48.0)	124 (52.0)	
Normal like	101 (51.0)	98 (49.0)	
**IntClustMemb**			
IntClustMemb 1	71 (51.0)	68 (49.0)	126.426 **(<0.001)**
IntClustMemb 2	33 (46.0)	39 (54.0)	
IntClustMemb 3	178 (61.0)	112 (39.0)	
IntClustMemb 4	145 (42.0)	198 (58.0)	
IntClustMemb 5	94 (49.0)	96 (51.0)	
IntClustMemb 6	52 (61.0)	33 (39.0)	
IntClustMemb 7	130 (68.0)	60 (32.0)	
IntClustMemb 8	221 (74.0)	78 (26.0)	
IntClustMemb 9	76 (52.0)	70 (48.0)	
IntClustMemb 10	80 (35.0)	146 (65.0)	
**Oestrogen Receptor (ER)**			
Negative	189 (43.0)	250 (57.0)	30.649 **(<0.001)**
Positive	869 (58.0)	629 (42.0)	
**Progesterone Receptor (PR)**			
Negative	432 (45.0)	517 (55.0)	65.768 **(<0.001)**
Positive	657 (63.0)	383 (37.0)	
**Human Epidermal Growth Factor Receptor 2** **(HER2)**			
Negative	963 (56.0)	770 (44.0)	5.863 **(0.015)**
Positive	117 (48.0)	130 (52.0)	

Significant *p* values are highlighted in bold.

**Table 2:** Associations between LAIR-1 protein expression, clinico-pathological features and other biomarkers in the whole cohort.

Parameters	LAIR-1 Cytoplasmic Expression		
	Negative/Low Expression N (%)	High Expression N (%)	*p* Value (χ2)
**Age at Diagnosis (years)**			
<50	101 (49.0)	105 (51.0)	0.898 (0.016)
≥50	180 (49.0)	183 (51.0)	
**Histological Grade**			
1	41 (54.7)	34 (45.3)	**<0.0001** (23.004)
2	115 (62.2)	70 (37.8)	
3	122 (40.3)	181 (59.7)	
**Stage**			
I	175 (51.0)	167 (49.0)	0.763 (1.158)
II	84 (47.0)	95 (53.0)	
III	19 (45.0)	23 (55.0)	
**Tumour Size**			
˂2.0 cm	125 (48.0)	133 (52.0)	0.657 (0.197)
≥2.0 cm	155 (50.0)	153 (50.0)	
**Histological Type**			
Ductal (including mixed)	225 (47.0)	256 (53.0)	**<0.001** (25.715)
Lobular	44 (79.0)	12 (21.0)	
Special type	10 (50.0)	10 (50.0)	
**IHC Subtypes**			
ER+/HER2-Low Proliferation	69 (61.6)	43 (38.4)	**0.019** (0.065)
ER+/HER2- High Proliferation	86 (49.4)	88 (50.6)	
Triple Negative	46 (42.2)	63 (57.8)	
HER2+	40 (43.0)	53 (57.0)	
**Nottingham Prognostic Index**			
GPG	88 (60.0)	59 (40.0)	**0.012** (8.866)
MPG	153 (47.0)	176 (53.0)	
PPG	39 (43.0)	51 (57.0)	
**Oestrogen (ER) Status**			
Negative	64 (41.0)	92 (59.0)	**0.013** (6.143)
Positive	216 (52.0)	194 (48.0)	
**Progesterone (PR) Status**			
Negative	112(47.0)	126 (53.0)	0.345 (0.891)
Positive	161 (51.0)	154 (49.0)	
**Human Epidermal Growth Factor Receptor 2 (HER2)**			
Negative	233 (50.0)	227 (50.0)	0.091 (1.113)
Positive	42 (45.0)	52 (56.0)	
**Epidermal Growth Factor Receptor (EGFR) (>10% as Positive Expression)**			
Negative	225 (51.0)	213 (49.0)	0.058 (3.604)
Positive	49 (42.0)	69 (58.0)	
**Phosphatidylinositol-4,5-Bisphosphate 3-Kinase, Catalytic Subunit Alpha (PIK3CA) (H-score > 100 as Positive Expression)**			
Negative	58 (57.0)	44 (43.0)	**0.048** (3.837)
Positive	160 (46.0)	189 (54.0)	
**Myc Proto-Oncogene Protein (c-MYC) (** **H-score > 35 as Positive Expression)**			
Negative	202 (55.0)	166 (45.0)	**<0.001** (27.38)
Positive	28 (26.0)	79 (74.0)	
**Cell Division Cycle 42 (Cdc42)** **(H-score > 150 as Positive Expression)**			
Negative	130 (57.0)	98 (43.0)	**<0.001** (16.24)
Positive	58 (36.0)	102 (64.0)	
**Ki67 (>10% as Positive Expression)**			
Negative	91 (57.0)	69 (43.0)	0.080 (3.071)
Positive	135 (48.0)	145 (52.0)	
**Cyclin B1 (>1% as Positive Expression)**			
Negative	113 (54.0)	97 (46.0)	**0.010** (6.630)
Positive	57 (40.0)	86 (60.0)	

Significant *p* values are highlighted in bold.

**Table 3:** Univariate and multivariate analysis of LAIR-1 expression for breast cancer-specific survival and distant metastasis-free survival.

Variable	Breast Cancer-Specific Survival						Distant Metastasis-Free Interval					
	Univariate			Multivariate			Univariate			Multivariate		
	HR	95% CI	*p* Value	HR	95% CI	*p* Value	HR	95% CI	*p* Value	HR	95% CI	*p* Value
**Whole Cohort**												
Tumour size	2.6	2.0–3.4	**<0.0001**	3.0	1.6–5.7	**0.0004**	2.3	1.8–2.8	**<0.0001**	2.3	1.4–3.9	**0.002**
Tumour stage	2.5	2.6–2.9	**<0.0001**	2.4	1.7–3.5	**<0.0001**	2.6	2.3–2.9	**<0.0001**	2.2	1.6–3.0	**<0.0001**
Grade	4.2	3.2–5.5	**<0.0001**	7.0	2.2–20.8	**0.001**	3.0	2.5–3.5	**<0.0001**	2.8	1.6–5.1	**<0.001**
CD8	0.7	0.5–1.0	0.084	0.5	0.3–1.1	**0.019**	0.9	0.7–1.2	0.470	0.9	0.4–1.8	0.673
CD3	1.0	0.7–1.6	0.851	1.1	0.5–3.0	0.7807	0.9	0.6–1.3	0.452	0.7	0.4–1.4	0.297
Her2+	3.1	2.4–4.1	**<0.0001**	2.1	1.2–3.5	**0.008**	2.7	2.1–3.5	**<0.0001**	1.8	1.1–3.1	**0.009**
Chemotherapy	2.1	1.6–2.8	**<0.0001**	0.9	0.5–1.7	0.880	2.0	1.6–2.6	**<0.0001**	0.7	0.4–1.2	0.172
**LAIR-1**	2.0	1.3–3.0	**0.002**	2.0	1.1–3.4	**0.017**	1.6	1.1–2.3	**0.013**	1.6	1.0–2.5	**0.046**
** LUMINAL A												
Tumour size	3.5	1.3–9.4	**0.013**	1.8	0.1–22.0	0.647	4.2	2.0–9.3	**0.0003**	7.3	0.7–17.0	0.088
Tumour stage	3.6	1.9–6.7	**<0.001**	3.4	0.3–33.0	0.292	3.0	1.8–4.8	**0.0001**	0.3	0.1–3.2	0.338
Grade	3.0	1.5–6.2	**0.003**	5.7	0.5–16.0	0.149	3.0	1.7–5.2	**0.0001**	1.1	0.3–3.6	0.890
Chemotherapy	1.8	0.2–13.6	0.580	2.9	0.2–41.0	0.414	2.3	0.6–10.0	0.250	2.2	0.2–28.0	0.557
**LAIR-1**	3.1	0.3–34.0	0.346	2.1	0.1–46.0	0.638	2.7	0.7–11.0	0.166	5.0	0.6–40.0	0.135
**LUMINAL B**												
Tumour size	1.8	1.1–2.9	**0.013**	1.7	0.6–5.2	0.337	2.1	1.5–3.2	**<0.001**	2.3	0.9–6.9	0.055
Tumour stage	2.5	1.8–3.3	**<0.0001**	1.2	0.4–3.1	0.788	2.3	1.8–3.0	**<0.0001**	1.0	0.4–2.3	0.989
Grade	3.1	1.9–5.2	**<0.0001**	10.1	1.3–75	**0.025**	2.2	1.6–3.1	**0.0001**	2.3	0.9–5.4	0.066
CD8	0.7	0.4–1.3	0.268	1.3	0.3–5.1	0.756	0.8	0.5–1.3	0.401	1.1	0.3–3.7	0.873
CD3	0.9	0.4–2.1	0.923	1.3	0.1–12	0.834	0.8	0.4–1.4	0.450	1.2	0.3–6.0	0.789
Chemotherapy	1.2	0.6–2.2	0.658	0.6	0.1–2.3	0.531	1.1	0.7–2.0	0.666	0.4	0.1–2.0	0.264
**LAIR-1**	1.7	0.7–4.1	0.247	1.7	0.6–5.5	0.331	1.1	0.5–2.1	0.859	1.1	0.4–2.8	0.770
**HER2+**												
Tumour size	2.8	1.7–4.8	**0.0001**	5.0	1.3–18.4	**0.016**	2.0	1.3–3.1	**0.003**	1.8	0.7–5.1	0.245
Tumour stage	2.5	1.9–3.4	**<0.0001**	7.3	3.0–18.0	**<0.0001**	2.4	1.8–3.2	**<0.0001**	7.0	3.2–15.0	**<0.0001**
Grade	2.0	0.9–4.1	**0.048**	3.3	0.4–27.0	0.261	1.5	0.9–2.6	0.167	0.6	0.1–1.9	0.311
CD8	0.6	0.4–1.2	0.143	0.1	0.1–0.5	**0.002**	0.6	0.4–1.1	0.126	0.4	0.2–0.6	0.113
CD3	0.9	0.3–2.7	0.944	2.2	0.3–15.0	0.405	0.7	0.3–1.6	0.427	2.4	0.4–16.3	0.369
Chemotherapy	1.5	0.9–2.5	0.144	2.6	0.9–7.0	0.070	1.3	0.8–2.2	0.232	0.9	0.4–2.3	0.866
**LAIR-1**	2.5	1.1–5.3	**0.014**	4.8	1.8–13.0	**0.003**	2.0	1.0–4.1	**0.044**	5.8	1.9–17.0	**0.002**
**TNBC**												
Tumour size	1.3	0.8–2.1	0.308	2.8	0.9–9.0	0.086	1.1	0.7–1.8	0.553	1.6	0.5–4.5	0.364
Tumour stage	2.0	1.5–2.7	**<0.0001**	3.2	1.6–6.5	**0.001**	2.0	1.5–2.6	**<0.0001**	2.8	1.4–5.2	**0.002**
CD8	0.5	0.2–0.8	0.011	0.3	0.1–1.3	0.194	0.5	0.3–1.0	0.061	0.4	0.1–1.7	0.222
CD3	0.5	0.2–0.10	0.050	0.7	0.1–5.6	0.764	0.5	0.2–1.1	0.077	1.3	0.2–9.2	0.815
Chemotherapy	0.9	0.6–1.5	0.741	0.5	0.2–1.3	0.145	0.9	0.5–1.3	0.431	0.5	0.1–1.3	0.163
**LAIR-1**	1.3	0.6–2.8	0.476	1.1	0.4–3.1	0.872	1.4	0.7–2.9	0.322	1.0	0.3–2.7	0.982

** In luminal A subtype CD3 and CD8 the number is too small, so it is not included in the model. Significant *p* values are highlighted in bold.

## Data Availability

3rd Party Data: Restrictions apply to the availability of these data. Data was obtained from [third party] and are available [from the authors/at URL] with the permission of [third party]. As Nottingham University Hospitals NHS Trust (NUH) stopped us from using the data unfortunately so we can’t provide the data without NUH permission.

## Supplementary Materials

The following are available online at https://www.mdpi.com/2072-6694/13/1/80/s1, Figure S1: Correlation of *LAIR-1* gene with other immune biomarkers using Breast Cancer Gene-Expression Miner v 4.5 (**A**) *CD3D*, (**B**) *CD8B*, (**C**) *FOXP3* and (**D**) *CD274(PD-L1).* Similarly, with TCGA database showing the association, with *LAIR-1* and (**E**) *CD3D*, (**F**) *CD8B*, (**G**) *FOXP3* and (**H**) *CD274(PD-L1).;* Figure S2: Kaplan-Meier Plots of LAIR-1 mRNA and patient outcome in breast cancer using the METABRIC cohort (**A**), and BC-gene miner database (**B**) All DNA microarray cohort. cBio Cancer Genomics Portal datasets showing (**C**) overall survival and (**D**) Disease Free Survival plots. Figure S3: Kaplan-Meier plots of BCSS (**A**) and DMFS (**B**) in the Nottingham Breast Cancer cohort showing LAIR-1 protein level expression in patients who received chemotherapy. Figure S4: Differential protein expression of LAIR-1 in breast cancer cell lines. (**A**) LAIR-1 protein levels were quantified by densitometry and normalized to β-Actin levels, showing increased LAIR-1 expression in MDA-MB 231 and SKBr3 cell lines. (**B**) Downregulation of LAIR-1 expression using two siRNAs: Both LAIR-1 siRNAs (s8048 and S8049), showed complete knockdown. LAIR-1 siRNA oligonucleotides were effective and showed almost complete loss of LAIR-1 protein expression; in (**C**) SKBr3 and (**D**) MDA-MB 231. Green and red bands represent LAIR-1 and the house-keeping Beta-Actin, respectively. Figure S5: Western blotting image showing a single band for LAIR-1 antibody at ~70 Kda. Green and red bands represent LAIR-1 and the house-keeping Beta-Actin, respectively.
