# Supplementary material for: The ITIM-Containing Receptor: Leukocyte-Associated Immunoglobulin-Like Receptor-1 (LAIR-1) Modulates Immune Response and Confers Poor Prognosis in Invasive Breast Carcinoma
Source: Cancers (Basel). 2020 Dec 30;13(1):80. doi: 10.3390/cancers13010080 (PMC7795350; doi:10.3390/cancers13010080)
Supplement: Supplementary file 1 [file cancers-13-00080-s001.pdf]

Supplementary Materials

The ITIM-Containing Receptor: Leukocyte-Associated Immunoglobulin-Like Receptor-1 (LAIR-1) Modulates Immune Response and Confers Poor Prognosis in Invasive Breast Carcinoma

Chitra Joseph, Mansour A. Alsaleem, Michael S. Toss, Yousif A. Kariri, Maryam Althobiti, Sami Alsaeed, Abrar I. Aljohani, Pavan L. Narasimha, Nigel P. Mongan, Andrew R. Green and Emad A. Rakha

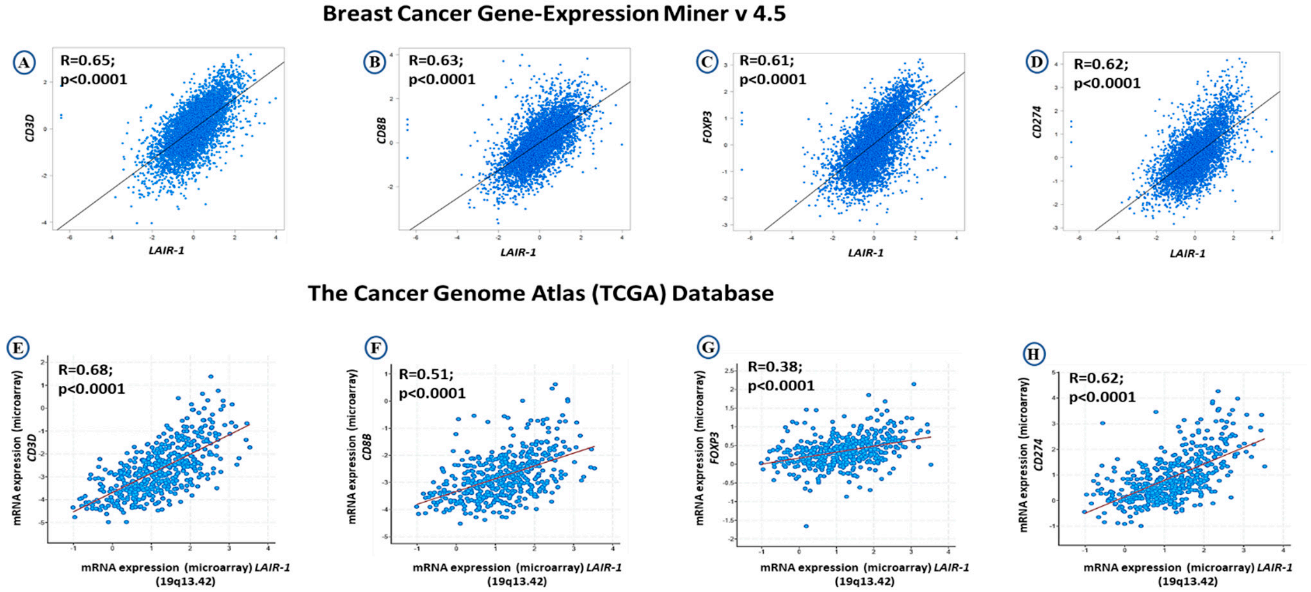

Figure S1. Correlation of *LAIR-1* gene with other immune biomarkers using Breast Cancer Gene-Expression Miner v 4.5 (**A**) *CD3D*, (**B**) *CD8B*, (**C**) *FOXP3* and (**D**) *CD274(PD-L1)*. Similarly, with TCGA data base showing the association, with *LAIR-1* and (**E**) *CD3D*, (**F**)*CD8B*, (**G**) *FOXP3* and (H) *CD274(PD-L1)*.

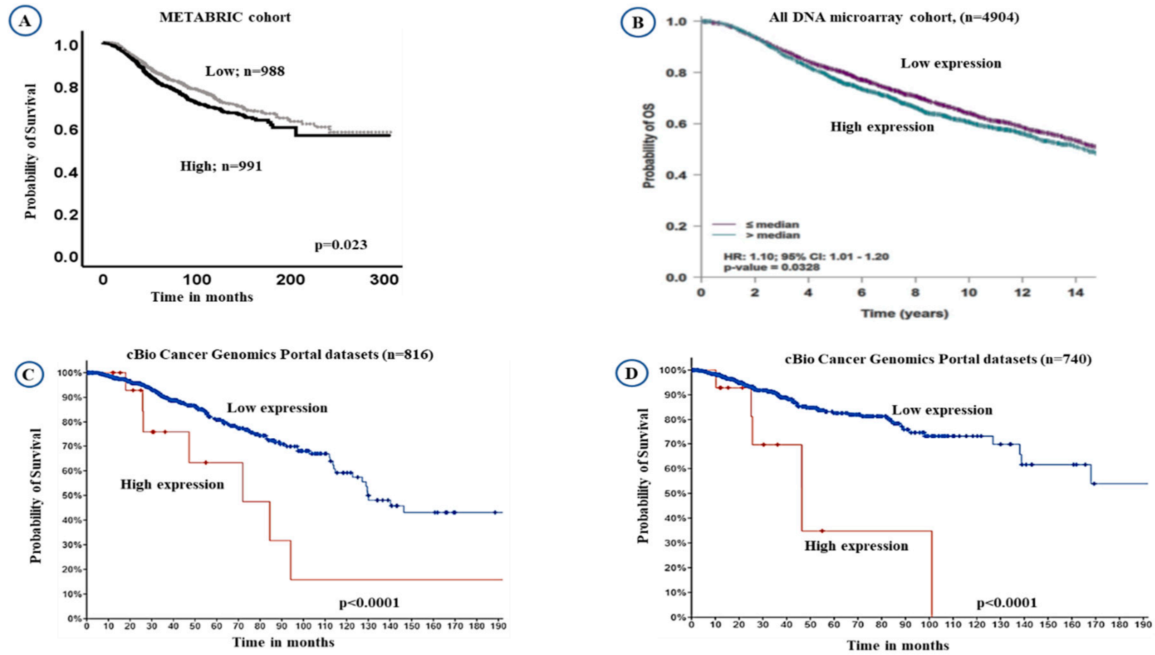

Figure S2. Kaplan–Meier Plots of *LAIR-1 mRNA* and patient outcome in breast cancer using the METABRIC cohort (**A**), and BC-gene miner database (**B**) All DNA microarray cohort. cBio Cancer Genomics Portal datasets showing (**C**) overall survival and (**D**) Disease Free Survival plots.

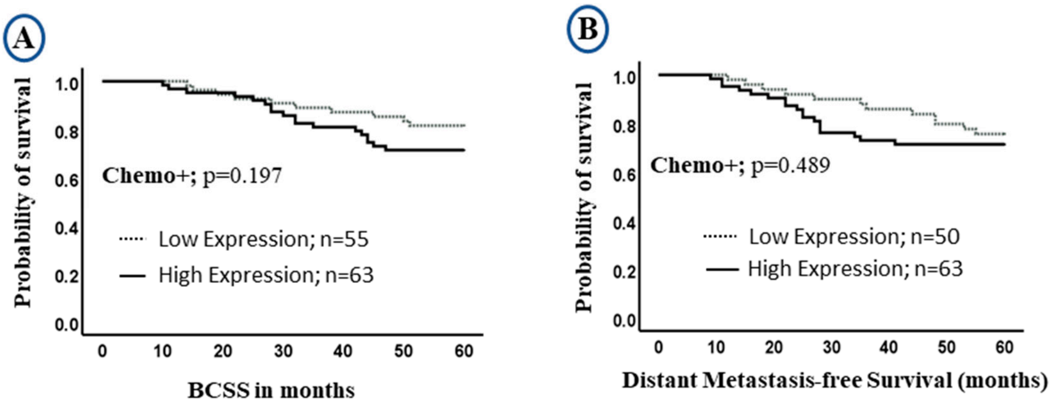

Figure S3. Kaplan–Meier plots of BCSS (**A**) and DMFS (**B**) in the Nottingham Breast Cancer cohort showing LAIR-1 protein level expression in patients who received chemotherapy.

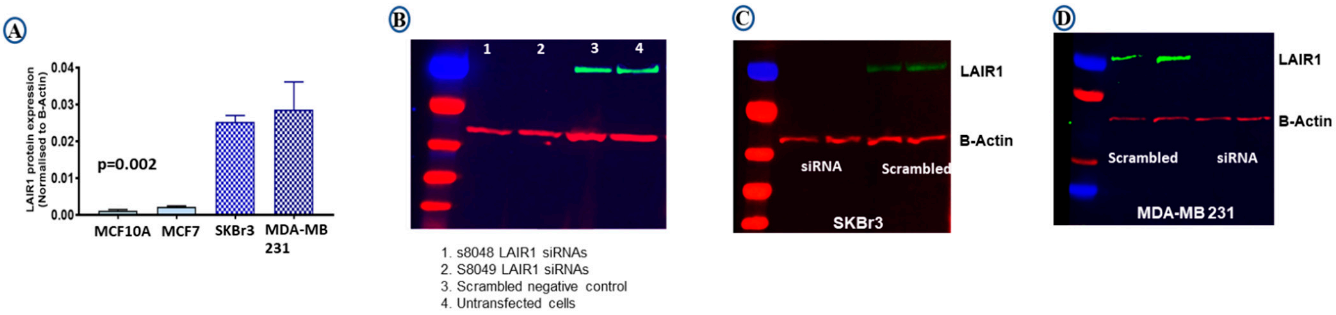

Figure S4. Differential protein expression of LAIR-1 in breast cancer cell lines. (**A**) LAIR-1 protein levels were quantified by densitometry and normalized to β-Actin levels, showing increased LAIR-1 expression in MDA-MB 231 and SKBr3 cell lines. (**B**) Downregulation of LAIR-1expression using two siRNAs: Both LAIR-1 siRNAs (s8048 and S8049), showed complete knockdown. LAIR-1 siRNA oligonucleotides were effective and showed almost complete loss of LAIR-1 protein expression; in (**C**) SKBr3 and (**D**) MDA-MB 231. Green and red bands represent LAIR-1 and the house-keeping Beta-Actin, respectively.

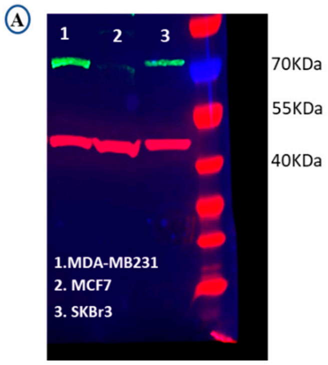

Figure S5. Western blotting image showing a single band for LAIR-1 antibody at ~70 Kda. Green and red bands represent LAIR-1 and the house-keeping Beta-Actin, respectively.
